# Correlation properties of heart rate variability during endurance exercise: A systematic review

**DOI:** 10.1111/anec.12697

**Published:** 2019-09-09

**Authors:** Thomas Gronwald, Olaf Hoos

**Affiliations:** ^1^ Department of Performance, Neuroscience, Therapy and Health MSH Medical School Hamburg Hamburg Germany; ^2^ Center for Sports and Physical Education Julius Maximilians University of Wuerzburg Wuerzburg Germany

**Keywords:** alpha1, autonomic nervous system, detrended fluctuation analysis, endurance exercise, heart rate variability, short‐term scaling exponent

## Abstract

**Background:**

Non‐linear measures of heart rate variability (HRV) may provide new opportunities to monitor cardiac autonomic regulation during exercise. In healthy individuals, the HRV signal is mainly composed of quasi‐periodic oscillations, but it also possesses random fluctuations and so‐called fractal structures. One widely applied approach to investigate fractal correlation properties of heart rate (HR) time series is the detrended fluctuation analysis (DFA). DFA is a non‐linear method to quantify the fractal scale and the degree of correlation of a time series. Regarding the HRV analysis, it should be noted that the short‐term scaling exponent alpha1 of DFA has been used not only to assess cardiovascular risk but also to assess prognosis and predict mortality in clinical settings. It has also been proven to be useful for application in exercise settings including higher exercise intensities, non‐stationary data segments, and relatively short recording times.

**Method:**

Therefore, the purpose of this systematic review was to analyze studies that investigated the effects of acute dynamic endurance exercise on DFA‐alpha1 as a proxy of correlation properties in the HR time series.

**Results:**

The initial search identified 442 articles (351 in PubMed, 91 in Scopus), of which 11 met all inclusion criteria.

**Conclusions:**

The included studies show that DFA‐alpha1 of HRV is suitable for distinguishing between different organismic demands during endurance exercise and may prove helpful to monitor responses to different exercise intensities, movement frequencies, and exercise durations. Additionally, non‐linear DFA of HRV is a suitable analytical approach, providing a differentiated and qualitative view of exercise physiology.

## 1 INTRODUCTION

In recent years, analytics conducted with non‐linear dynamics and the chaos theory have been adapted to gain further insights into the complex cardiovascular regulation during acute exercise bouts (Hottenrott & Hoos, 2017; Michael, Graham, & Davis, 2017). Thus, measures of the non‐linear dynamics of physiologic variability of heart rate (HR) time series, such as heart rate variability (HRV), may provide new opportunities to monitor cardiac autonomic regulation during exercise. Present research suggests that cardiac dynamics are controlled by complex interaction effects between the sympathetic and parasympathetic branches of the autonomic nervous system on the sinus node, and non‐neural factors (Persson, 1996). These two branches act competitively, resulting in clear sympathetic activation and parasympathetic withdrawal during exercise (Sandercock & Brodie, 2006). Evaluations of absolute HRV values of the time (e.g., SDNN, RMSSD) and frequency domain (e.g., LF, HF, LF/HF) show that exercise may diminish variability even at low to moderate exercise intensities to such an extent, that these measures may not be able to discriminate between a further increase in exercise intensity due to low signal‐to‐noise ratio (e.g., Casadei, Cochrane, Johnston, Conway, & Sleight, 1995; Hautala, Makikallio, Seppanen, Huikuri, & Tulppo, 2003; Sandercock & Brodie, 2006; Tulppo, Makikallio, Takala, Seppänen, & Huikuri, 1996). Consequently, findings derived from such linear parameters have led to inconsistent results during different exercise intensities (Hottenrott, Hoos, & Esperer, 2006; Sandercock & Brodie, 2006).

In healthy individuals, the HRV signal is mainly composed of quasi‐periodic oscillations, but it also possesses random fluctuations and so‐called fractal structures (Goldberger et al., 2002). Analysis of these structures has become a popular tool that is useful in the investigation of age and disease (Voss, Schulz, Schroeder, Baumert, & Caminal, 2009). In this respect, analysis methods of non‐linear dynamics in HRV do not describe the amplitude of the variability, but rather the qualitative characteristics of the structure, dynamics of the signal, and interaction of subsystems (Aubert, Seps, & Beckers, 2003; Mansier et al., 1996). One widely applied approach to investigate scaling characteristics is the detrended fluctuation analysis (DFA). This analysis provides a differentiated view on the correlative structure of variability caused by physiological processes in the time series (Goldberger et al., 2002; Hottenrott & Hoos, 2017). Thus, the DFA is a non‐linear method to quantify the fractal scale and the degree of correlation properties with an HRV signal in the form of a dimensionless measurement. The DFA has been referred to as a modification of the root mean square analysis (RMS) that is also suitable for analyzing short and non‐stationary time series data (Peng, Havlin, Stanley, & Goldberger, 1995). Briefly, the root mean square fluctuation of the integrated and detrended data is measured in observation windows of different sizes. The data are then plotted against the size of the window on a log–log scale. The scaling exponent represents the slope of the line, which relates (log) fluctuation to (log) window size (Mendonca et al., 2010).

It should be noted that the short‐term scaling exponent alpha1 of DFA (window width: 4 ≤ *n* ≤ 16 beats) has already been applied to cardiovascular risk assessment as well as prognosis and prediction of mortality in clinical settings (Huikuri, Perkiömäki, Maestri, & Pinna, 2009; Peng et al., 1995; Platisa & Gal, 2008; Sen & McGill, 2018; Vanderlei, Pastre, Júnior, & Godoy, 2010) and is also suitable for applied sport‐specific settings including higher exercise intensities, non‐stationary data, and relatively short recording times for different conditions (Hautala, Kiviniemi, & Tulppo, 2009; Hautala et al., 2003; Tulppo et al., 2001). The current body of research in this field during resting state shows that regardless of the disease or age group investigated, values of alpha1 that differ from the normal value of approximately 1.0 (decreasing or increasing) are associated with higher morbidity or worse prognosis (Huikuri et al., 2009; Sen & McGill, 2018). This indicates a loss of fractal dynamic toward a more random (disorganized randomness) or a more strongly correlated (periodicity) behavior (de Godoy, 2016).

In a healthy resting state, the fractal dynamics of HRV may be related to the maintenance of basic stability of the control systems between order (persistence) and disorder (change) in the context of a homeodynamic approach and generalized regulation mechanism for organisms (Iyengar, Peng, Morin, Goldberger, & Lipsitz, 1996; Kauffman, 1995; Makikallio et al., 1999; Yates, 1994). This is one mechanism by which a complex biological network may not only be able to avoid too much order, but also too much chaos, to remain close to a critical threshold under resting conditions. When physiologic systems lose their fractal complexity, they are less adaptable and less able to cope with varied stimuli such as exposure to different modes of exercise or changing environmental conditions (Goldberger, 1997). Currently, there is only scarce evidence for risk stratification during exercise; however, it is rather the qualitative information of organismic regulation during exercise that seems to be important in order to gain new insights from a dose–response perspective and for the application of exercise and training prescription in training and therapy interventions (Gronwald et al., 2019a). The quality of regulation at rest and during exercise may reflect a new possibility to control training in health care, therapy, or elite sports. As the DFA is very robust against artefacts and has a low dependence on HR (Peng et al., 1995; Sandercock & Brodie, 2006), this method seems to be suitable for analyzing the complexity of cardiac autonomic regulation during various exercise intensities, modalities, and environmental conditions (Hottenrott & Hoos, 2017; Gronwald et al., 2019a).

Therefore, the purpose of this article was to systematically review the literature on the changes in the short‐term scaling exponent alpha1 of the DFA as a proxy of correlation properties in the HR time series during acute endurance exercise.

## 2 METHODS

### 2.1 Search strategy

The systematic literature review followed the established guidelines of Moher, Liberati, Tetzlaff, and Altman (2009) and Wright, Brand, Dunn, and Spindler (2007). The systematic literature search was conducted independently by the first and last author of this article in May 2019 using the online databases PubMed and Scopus. The request consists of three search fields with independent search terms. The search fields were connected with “AND” in order to ensure that at least one of the terms can be found in the results. All terms in one search field were linked with the conjunction “OR.” The first group comprised the measures heart rate and heart variability. The second search field comprised the method of detrended fluctuation analysis. The third search field comprised possible synonyms for exercise and physical exertion. The search terms were (“heart rate variability” OR HRV OR “heart beat variability” OR “heart rate” OR HR OR “heart beat”) AND (“detrended fluctuation analysis” OR DFA OR “short‐term scaling” OR “short term scaling” OR “short‐term scaling exponent” OR “short term scaling exponent” OR alpha1 OR “correlation properties” OR fractals OR “fractal dynamics” OR “fractal correlation” OR “fractal correlation properties” OR dynamics OR “non‐linear dynamics” OR “non linear dynamics” OR “autonomic activity” OR “cardiac autonomic activity” OR “autonomic nervous system activity”) AND (exercise OR “physical exertion” OR “physical activity” OR “physical stress” OR “physical strain” OR “physical demand”). We used the integrated filter systems of the databases to minimize the amount of irrelevant studies. As filters within the databases, “abstract availability,” “publication date > 1995,” and “human subjects” (not available in Scopus) were used. In PubMed, the research was undertaken only in the categories title and abstracts, whereas in Scopus a restriction was only available for abstracts.

### 2.2 Study selection and data extraction

Studies with cross‐sectional and longitudinal design, as well as exercise intervention studies, were eligible for this review. Eligible studies had to (a) investigate HRV and short‐term scaling exponent alpha1 of DFA (window width: *n* ≤ 16 beats) during acute exercise bouts (dynamic endurance exercise), (b) be published in peer‐reviewed journals in English or German language (conference abstracts, dissertations, theses and book chapters were not included), and (c) focus on healthy human subjects. Studies were excluded, (a) if subjects were recruited from non‐healthy populations, (b) if there were only analysis under resting conditions or passive recovery, (c) if there was a different study or non‐comparable method focus, (d) if there was insufficient information about the used exercise variables, (e) if there was no analysis of dynamic endurance exercise, or (f) if medication and/or other interventions were tested during exercise. Since the focus of this review is on the effects of acute bouts of exercise on short‐term scaling exponent alpha1 of DFA, we further excluded intervention studies investigating DFA only under rest conditions. No age‐ or gender‐specific restrictions were imposed in this review.

A spreadsheet was used to include the extracted data. After merging search results, discarding duplicates, and screening of titles and abstracts, full texts of the remaining studies were reviewed with regard to inclusion and exclusion criteria. Additionally, reference lists of available articles and contents of relevant journals were reviewed. If abstracts of studies met the inclusion criteria, but full texts were not available, or if data necessary for the review could not be found in the article, corresponding authors were contacted. For studies with more than one article based on the same study population, inclusion was limited to the original publication. From each article included in the review, the following relevant data were extracted and reported: author, year of publication, population characteristics (sample size, age, gender, performance level (if available)), study design (exercise mode), type of HRV measurement method, and main outcomes.

## 3 RESULTS

**Figure 1:**
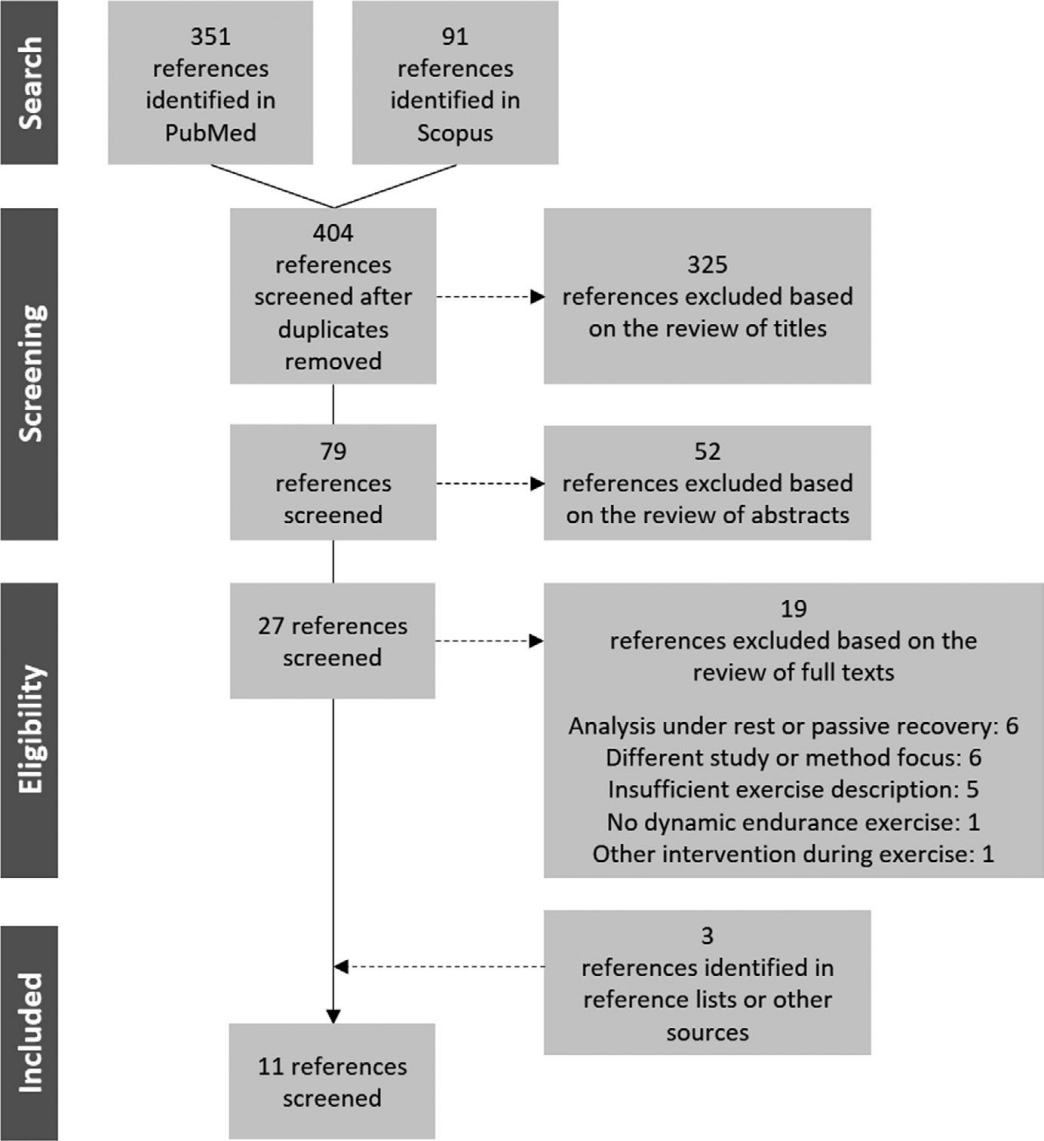
Systematic literature search scheme with numbers of included and excluded studies

The initial search using the mentioned keywords identified 442 records (351 in PubMed, 91 in Scopus). After the process of removal of duplicate articles (*n* = 38), a total of 404 articles remained. 377 articles were removed by title or abstract, remaining a total of 27 articles. In addition, 19 articles were removed after full‐text screening with reason: analysis under rest or passive recovery (Blasco‐Lafarga, Martínez‐Navarro, & Mateo‐March, 2013; Heffernan et al., 2008; Mendonca et al., 2010; Millar, MacDonald, Bray, & McCartney, 2009; Millar, Rakobowchuk, McCartney, & MacDonald, 2009; de Rezende Barbosa et al., 2018), different study or method focus (Bardet, Kammoun, & Billat, 2012; Billat, Mille‐Hamard, Meyer, & Wesfreid, 2009; Billat, Wesfreid, Kapfer, Koralsztein, & Meyer, 2006; Boullosa, Barros, Rosso, Nakamura, & Leicht, 2014; Castiglioni, Quintin, Civijian, Parati, & Rienzo, 2007; Perkins, Jelinek, Al‐Aubaidy, & Jong, 2017), insufficient description of exercise variables (Karasik et al., 2002; BuSha 2010; Chen, Liaw, Chang, Chan, & Chiu, 2015; Chen, Liaw, Chang, Chuang, & Chien, 2015; Bernaola‐Galván, Gómez‐Extremera, Romance, & Carpena, 2017), no dynamic endurance exercise (Zhuang et al., 2008), and other intervention during exercise (Weippert, Behrens, Rieger, Kumar, & Behrens, 2015). Two articles were added because they were identified in reference lists, one article was added from our own study group. A summary of the search according to the PRISMA guidelines (Moher et al., 2009), including the number of studies suitable for qualitative synthesis, is shown in Figure 1. The 11 studies included for review are summarized in Table 1.

**Table 1:** Summary of included studies

Study	Sample	Study design	Measures	Results of DFA‐alpha1
Tulppo et al. (2001)	20 male volunteers (29 ± 5yr)	30min prolonged walking exercise (low intensity: 4km/h)	Polar R‐R recorder + continuous‐surface TEC−7100 electrocardiogram; last 500 beats during prolonged exercise; measures: HR, LF, HF, TP, ApEn, DFA‐alpha1 (window width: 4 ≤ *n* ≤ 11 beats), BP	Prolonged low intensity exercise versus baseline: ↑
Hautala et al. (2003)	9 male volunteers (37 ± 11yr)	Incremental cycling exercise test until voluntary exhaustion (start: 50W for 5min, increment: 25W/3min); 20min prolonged walking/running exercise (low intensity: 4km/h; mid intensity: 12km/h)	Polar real‐time microprocessor QRS detector system; 3min intervals during incremental test, 15min during prolonged exercise; measures: HR, SDNN, LF, HF, DFA‐alpha1 (window width: 4 ≤ *n* ≤ 11 beats)	Biphasic course with increasing exercise intensity in the incremental test: ↑↓; Prolonged low intensity exercise: ↑; Prolonged mid intensity exercise: ↓
Casties et al. (2006)	7 endurance trained male cyclists (22.9 ± 2.5yr)	Incremental cycling exercise test (3 stages: 8min at 40% VO_2MAX_ from an incremental test, 8min at 70%, 8min at 90%), 50min recovery (sitting)	Ela medicals electrocardiogram; 5min intervals during exercise, 10min during recovery; measures: meanRR, SDNN, RMSSD, LF, HF, LLE, DFA‐alpha1 (window width: 4 ≤ *n* ≤ 16 beats)	Biphasic course with increasing exercise intensity: ↑↓; Recovery: ↑
Platisa and Gal (2008)	10 male volunteers (22.8 ± 2.4yr)	Incremental running exercise test until voluntary exhaustion (start: 9km/h and incline of 2%, increment: 2%/3min), 15min recovery (supine)	Viasys electrocardiogram; last stage of running, 3rd min of recovery; measures: meanRR, SDNN, DFA‐alpha1 (window width: *n* < 11 beats), DFA‐alpha2 (window width: *n* ≥ 11 beats)	Last stage versus baseline: ↓; Recovery: ↑
Platisa et al. (2008)	9 trained male basketball players (18.0 ± 0.7yr), 11 untrained male volunteers (22.8 ± 0.7yr)	Incremental running exercise test until voluntary exhaustion (start: 9km/h and incline of 2%, increment: 2%/3min), 15min recovery (supine)	Viasys electrocardiogram; last stage of running, 5th min of recovery; measures: HR, LF, HF, DFA‐alpha1 (window width: 4 ≤ *n* ≤ 16 beats), SampEn, BF, VO_2_, RER	Last stage versus baseline ‐ in both groups: ↓ (Pooled data: biphasic course with increasing exercise intensity in the incremental test: ↑↓); Recovery: ↑
Karavirta et al. (2009)	93 male volunteers (55.6 ± 7.4yr)	Incremental cycling exercise test until voluntary exhaustion (start: 50W, increment: 20W/2min, cadence: 60rpm)	Polar s810i; 2min intervals; measures: HR, LF, HF, DFA‐alpha1 (window width: 4 ≤ *n* ≤ 11 beats), VO_2_	Biphasic course with increasing exercise intensity: ↑↓
Blasco‐Lafarga et al. (2017)	13 endurance trained male cyclists (15.43 ± 0.51yr)	Incremental cycling exercise test until voluntary exhaustion (start: 10min of warm‐up pedaling: ≈ 50% VO_2MAX_ (< 120W, interspersed with 3 submaximal self‐selected accelerations of 10 to 20s), increment: 30W/4min, every stage followed by 30s for blood lactate collection (allowed to slow down), cadence: self‐selected)	Polar RS800; 3min intervals; measures: HR, meanRR, RMSSD, SD1, DFA‐alpha1 (window width: 4 ≤ *n* ≤ 16 beats), VO_2_, RER, BL, SaO_2_, RPE	Increasing exercise intensity: ↓
Gronwald et al. (2018)	16 endurance trained male cyclists (25.9 ± 3.8yr)	60min prolonged cycling exercise at 90% IANS from an incremental test; varied cadences [rpm] every 10 min (90(1), 120(1), 60(1), 120(2), 60(2), 90(2)), 10min recovery (100W)	Polar s810i; 2min intervals; measures: HR, meanRR, SDNN, DFA‐alpha1 (window width: 4 ≤ *n* ≤ 16 beats), BL, RPE	90(1) versus 120(1): ↓; 60(1) versus 120(2): ↓; 90(1) versus 90(2): ↓; Recovery: ↑
Gronwald et al. (2019)	16 endurance trained male cyclists (25.9 ± 3.8yr)	Incremental cycling exercise test until voluntary exhaustion (start: 100W, increment: 20W/3min, cadence: 80−90rpm)	Polar s810i; 2min intervals; measures: HR, meanRR, SDNN, RMSSD, DFA‐alpha1 (window width: 4 ≤ *n* ≤ 16 beats), VO_2_, RER, BL, RPE	Biphasic course with increasing exercise intensity: ↑↓
Gronwald et al. (2019a)	9 endurance trained male cyclists (26.4 ± 4.1yr)	Prolonged cycling exercise at IANS from an incremental test until voluntary exhaustion (10%–100%)	Polar s810i; 2min intervals; measures: HR, meanRR, SDNN, DFA‐alpha1 (window width: 4 ≤ *n* ≤ 16 beats), SpO_2_, BL, RPE	10% versus 100%: ↓; Recovery: ↑
Gronwald et al. (2019b)	16 endurance trained male cyclists (25.9 ± 3.8yr)	Interval cycling session (3(IB)x5 intervals with 60s at P_MAX_ from an incremental test; 60s recovery between intervals and 10min (AR) after each IB at 100W, cadence: 80−90rpm)	Polar s810i; 1min intervals; measures: HR, meanRR, RMSSD, DFA‐alpha1 (window width: 4 ≤ *n* ≤ 16 beats), BL, RPE	IB versus AR: ↑; AR versus IB: ↓; no change during the course of AR

Abbreviations: ApEn, Approximate entropy; AR, Active recovery; BF, Breathing frequency; BL, Blood lactate concentration; BP, Blood pressure; DFA‐alpha1, Short‐term scaling exponent of detrended fluctuation analysis; DFA‐alpha2, Long‐term scaling exponent of detrended fluctuation analysis; HF, High‐frequency band; HR, Heart rate; IANS, Individual anaerobic threshold; IB, Interval block; LF, Low‐frequency band; LLE, Largest Lyapunov Exponent; meanRR, Average of normal R‐R intervals; RER, Respiratory exchange ratio; RMSSD, root mean square of successive differences; RPE, rate of perceived exertion; SampEn, Sample entropy; SDNN, Standard deviation of all normal RR‐intervals; SD1, Transversal axis from the Poincare plot; SpO_2_, Oxygen saturation of the blood; TP, Total power; VO_2_, oxygen uptake. ↑: Increase of DFA‐alpha1, ↓: Decrease of DFA‐alpha1.

In all studies, only men were examined, mostly in early to middle adulthood. Only Blasco‐Lafarga, Camarena, and Mateo‐March (2017) analyzed elite youngster cyclists of approx. 15 years and Karavirta et al. (2009) older untrained men aged 40–67 years. Six studies included endurance trained cyclists, one study well‐trained basketball players, and the others untrained volunteers. According to the study design, seven of the included studies used an incremental cycling exercise test with different starting wattage, increment and stage duration until voluntary exhaustion (Blasco‐Lafarga et al., 2017; Casties, Mottet, & Gallais, 2006; Gronwald, Hoos, Ludyga, & Hottenrott,^2019^; Hautala et al., 2003; Karavirta et al., 2009; Platisa & Gal, 2008; Platisa, Mazic, Nestorovic, & Gal, 2008), four of the studies used a prolonged exercise regime with low‐intensity walking of 30 and 20 min (Hautala et al., 2003; Tulppo et al., 2001), mid‐intensity running exercise of 20 min (Hautala et al., 2003), mid‐intensity cycling exercise of 60 min (Gronwald, Ludyga, Hoos, & Hottenrott, 2018), and mid‐intensity cycling exercise corresponding to the individual anaerobic threshold until voluntary exhaustion (Gronwald et al., 2019a). In addition, Gronwald et al. (2018) investigated the influence of varied cadences during prolonged cycling exercise. Only one study examined a short‐term cycling interval session which alternated with active recovery periods (Gronwald et al., 2019b).

## 4 DISCUSSION

The aim of this review was to analyze studies that investigated the effects of acute endurance exercise on the short‐term scaling exponent alpha1 of the DFA as a proxy of correlation properties in the HR time series. Of the 442 articles found in the databases and additional sources, 11 met the inclusion criteria.

### 4.1 Influence of exercise intensity

In the studies investigating incremental cycling exercise tests until voluntary exhaustion, the results indicate a time‐dependent loss of variability and complexity of R‐R interval fluctuations with increasing exercise intensity, independent of different starting wattage, increment, and stage duration (Blasco‐Lafarga et al., 2017; Casties et al., 2006; Gronwald, Hoos & Hottenrott, 2019a; Gronwald et al., 2019; Hautala et al., 2003; Karavirta et al., 2009; Platisa & Gal, 2008; Platisa et al., 2008). In addition, in the studies analyzing different intervals of incremental tests from low to high‐intensity exercise stages, DFA‐alpha1 develops a biphasic course (with a plateau according to low exercise intensity) with increasing exercise intensity (Casties et al., 2006; Gronwald et al., 2019; Hautala et al., 2003; Karavirta et al., 2009; Platisa et al., 2008). Depending on the resting value in healthy states (DFA‐alpha1 usually around 1.0), stable or slightly rising values of DFA‐alpha1 up to 1.5 have been reported at very low to mild intensities, indicating a strongly correlated structure of HR dynamics due to vagal withdrawal. Conversely, from moderate to high exercise intensity, DFA‐alpha1 decreased almost linearly and showed high negative correlations with the rising rating of perceived exertion and oxygen uptake until voluntary exhaustion (DFA‐alpha1 usually < 0.5) (Blasco‐Lafarga et al., 2017; Gronwald et al., 2019). Therefore, DFA‐alpha1 seems to be suitable to distinguish between different organismic demands and may prove helpful to monitor responses to different exercise intensities until voluntary exhaustion (Gronwald et al., 2019; Hautala et al., 2003). This appears to be an advantage over standard HRV time and frequency domain parameters, since they are usually strongly amplitude‐dependent and limited in their informative value under moderate to high exercise intensities (Sandercock & Brodie, 2006).

The described behavior may indicate a qualitative change in the self‐organized regulation of cardiac rhythm, which coincides with the gradually increasing HR (Platisa et al., 2008). This may be related to the maintenance of basic stability of the control systems between order and disorder in the context of homeodynamics under resting conditions and moderate exercise intensity (Kauffman, 1995). In this context, a characteristic of a complex biological network seems to be the ability to not only avoid too much order, but also too much chaos, or to stay close to a critical threshold under resting states. The strongly correlated structure of HR dynamics during low to mild exercise intensity could be due to a neutral state of the primary HRV modulators, which might happen when the intrinsic heart rate (IHR) is reached (Gronwald et al., 2019; Platisa et al., 2008). In this state, neither the sympathetic nor the parasympathetic nervous system has to modulate autonomic sinus node activity, so that the intrinsic characteristic of the cardiomyocytes in the sinus node could cause this correlation of the HRV signal (Jose & Collison, 1970; Opthof, 2000; Platisa et al., 2008; Stein, Medeiros, Rosito, Zimerman, & Ribeiro, 2002). The subsequent gradual decrease of DFA‐alpha1 with increasing exercise intensity indicates an intensity‐dependent change from strongly correlated to uncorrelated/stochastic or anti‐correlated behavior of the R‐R intervals (Platisa & Gal, 2008). Additionally, one study examined a short‐term cycling interval session with high exercise intensity alternated with active recovery periods (Gronwald et al., 2019b). The data show a decrease in DFA‐alpha1, with an increasing exercise intensity during the interval blocks. DFA‐alpha1 values return to the level of the warm‐up periods very quickly during light active recovery and remain nearly the same until the end of the active recovery phase.

In the past, these changes in correlation properties of HR dynamics during exercise have been explained by a random walk model with some kind of stochastic feedback (Ivanov, Nunes Amaral, Goldberger, & Stanley, 1998). The loss of complexity of the HR time series during exercise is related to the disruption of the equilibrium between the two branches of the autonomic nervous system, due to the decreased parasympathetic activity and/or the increased sympathetic activity (Sandercock & Brodie 2006; Lewis & Short, 2010). This particular change could be due to organismic system withdrawal, which aims to protect homeodynamic processes (Casties et al., 2006; Platisa et al., 2008) that are matched by the central autonomic network (CAN) as an anatomical structure of the central nervous system integrating various internal and external stimuli (Benarroch, 1993, 1997). In addition to these influences, the great loss of correlation properties might be a consequence of complementary neural mechanisms/circuits (Shaffer, McCraty, & Zerr, 2014) to maintain locomotor‐respiratory coupling during cycling in the context of coordination between heartbeat, breathing patterns, and movement frequency (e.g., cadence in cycling exercise) (Blasco‐Lafarga et al., 2017; Casties et al., 2006; Gronwald et al., 2018).

Another possible explanatory approach for the decrease in HRV complexity during endurance exercise could be an increased reduction in the input number of different physiological systems and/or that in the interaction of various subsystems, with a particular focus on one dominant system or a few dominant systems (Casties et al., 2006; Nakamura, Yamamoto, & Muraoka, 1993). This could be interpreted in the sense of centralization or “mechanization” of a complex physiological system (von Bertalanffy, 1950) and indicate a restriction of cardiovascular self‐regulation, which reduces the adaptability to further perturbations and ultimately endangers the integrity of the overall system. In the sense of this mechanization of the organismic regulation, a dominant “performance attractor” could emerge during high physiological demands, which could be determined by sympathetic activity (Hautala et al., 2003; Karasik et al., 2002), neuro‐mechanical coupling of several oscillators (Casties et al., 2006), and/or the non‐neural, intrinsic HR regulation (Platisa & Gal, 2008). Thus, every fluctuation is corrected immediately in the opposite direction by the dominant attractor (Karasik et al., 2002), which results in a random or anti‐correlated signal. This organismic system withdrawal may also be interpreted as a loss of systemic integrity, in the sense of a hazardous situation for homeostasis (Seely & Macklem, 2004), which may only be tolerated for a short time period.

In addition to the included studies in this review, Hottenrott and Hoos (2017) disclosed some unpublished data in their book chapter showing differences in the time course of DFA‐alpha1 during incremental cycling exercise in athletes of three different aerobic fitness levels (high: VO_2_peak: 58.7 ± 5.4 ml min^−1^ kg^−1^, *n* = 18; medium: VO_2_peak: 52.3 ± 5.4 ml min^−1^ kg^−1^, *n* = 15; low: VO_2_peak: 41.9 ± 6.8 ml min^−1^ kg^−1^, *n* = 16). The presented data extend the findings of this review concerning exercise intensity as there is a gradual decrease of alpha1 during graded exercise, denoted by significant changes compared to the previous intensity level, whereby degree and progression of uncorrelated HR dynamics significantly differ between trained and untrained subjects. Additionally, a crossover phenomenon may be present, as with intensities above 70% VO_2_peak, both trained groups (medium and high level) show a more pronounced reduction in DFA‐alpha1 compared to the untrained state, similar to the findings of Platisa et al. (2008). Further research is needed to elucidate the physiological mechanisms underlying these differences in the trained state.

### 4.2 Interacting factors for exercise intensity: exercise duration and cadence

Data on the direct influence of exercise duration on DFA‐alpha1 are scarce. Only two studies used a prolonged exercise regime with repeated HRV measures during low‐intensity walking for 30 and 20 min and found an increase in DFA‐alpha1 (Hautala et al., 2003; Tulppo et al., 2001). On the other hand, another three studies that investigated a prolonged exercise regime with medium intensity including 20 min of running, 60 min of cycling exercise and cycling exercise corresponding to the individual anaerobic threshold until voluntary exhaustion found a substantial decrease in DFA‐alpha1 (Gronwald et al., 2018; Hautala et al., 2003; Gronwald et al., 2019a). Taken together, these findings support the notion that exercise intensity and duration may have an interacting effect on DFA‐alpha1 during exercise. It seems that prolonged exercise with low‐intensity reintegrates and synchronizes the subsystems, while prolonged exercise with mid‐ to high‐intensity disintegrates and mechanizes the whole system, which is only tolerable for a limited period of time. Beyond the objective of this review, it should also be mentioned that Gronwald et al. (2019a) additionally analyzed the influence of normobaric hypoxia compared to normoxia during the prolonged exercise until voluntary exhaustion. The data showed that hypoxia provoked higher demands and loss of correlation properties (decrease of DFA‐alpha1) at an earlier stage during the exercise regime compared to normoxia, implying an accelerated alteration of cardiac autonomic regulation.

Furthermore, Gronwald et al. (2018) investigated the influence of varied cadences during the prolonged cycling regime of 60 min. The study indicates that the assessment of DFA‐alpha1 allows a distinction between varied cadences with decreasing values accompanied by an increasing cadence and prolonged duration. The interacting influence of cadence and exercise duration (constant workload and cadence of 90 rpm at the beginning and end of the prolonged exercise) verifies a demand‐dependent change from strongly correlated to uncorrelated/stochastic or anti‐correlated behavior of the R‐R intervals (Gronwald et al., 2018; Platisa & Gal, 2008) quite similar to the RR dynamics during high‐intensity exercise.

Summing up the aforementioned evidence on DFA‐alpha1 of HR dynamics during endurance exercise, this approach might offer new perspectives for the evaluation of complex models of exercise fatigue and endurance performance (Abbiss & Laursen 2005; Marcora, 2008; Ament & Verkerke 2009; Noakes, Gibson, & Lambert, 2004; Millet, 2011; Noakes, 2011, 2012; St Clair Gibson, Swart, & Tucker, 2018) and may shed new light on the concept of cardiac control during exercise that focuses on a so‐called “central command” (Boulpaep, 2009; Williamson, 2010, 2015; Williamson, Fadel, & Mitchell, 2006). Non‐linear HRV analysis with DFA might be a suitable approach for this purpose. The complex integration of peripheral and central information on the self‐organized down regulation and limitation of muscle recruitment as a protection of the organismic homeostasis postulated by Noakes et al. (2004) may be explored further.

## 5 LIMITATION

Despite the difference in protocols (exercise type, intensity, duration, cadence, environmental conditions) of the included studies, the application of DFA might help analyze the relationship between different modes of exercise and the corresponding altered cardiac autonomic regulation. This could help overcome the limitations of strongly decreased variability and weak reproducibility of the frequency‐domain HRV measures employed during exercise (Millar et al., 2009; Persson & Wagner, 1996; Tulppo et al., 2005). Further studies are necessary to prove the reliability of DFA‐alpha1 especially during high‐intensity exercise. Only the study by Boullosa et al. (2014) has demonstrated the high relative and absolute reliability of DFA‐alpha1 during light intense walking (before and after maximal efforts) and the usefulness of this measure for evaluating autonomic responses during constant submaximal exercises. Additionally, the results of Boullosa et al. (2014) confirm that the short‐term components (e.g., RMSSD, SD1, and DFA‐alpha1) were more reliable (greater ICC) than long‐term components of HRV (e.g., SDNN, SD2, and DFA‐alpha2). To gain further insights in terms of the age‐ (Iyengar et al., 1996; Voss et al., 2009) and gender‐specific (Mendonca et al., 2010) behavior of DFA‐alpha1 during endurance exercises, further investigations with participants of both sexes across all ages (a greater age‐range) are necessary. In addition, we are aware that human physiology and cardiac autonomic regulation during exercise is too complex and too dependent on certain conditions and assumptions to be broken down into a single key measure. Therefore, further methods, such as multifractal or multiscale analytics, are currently being developed to enable in‐depth analysis information pertaining to cardiac autonomic control during exercise. Multifractal characterization appears to be a useful method for exploring the physiological basis of long‐term correlation structure in HR time series in the context of exercise and training (Castiglioni & Faini, 2019; Lewis & McNarry, 2013). Nevertheless, non‐linear analysis of HRV with DFA‐alpha1 promises a differentiated and qualitative view of acute (and may be chronic adaptational) exercise responses and seems to be applicable in combination with other internal and external load measures used in diagnostics, monitoring, and training control (during resting states and during standardized exercise settings).

## 6 CONCLUSION

DFA‐alpha1 of HRV is suitable to distinguish between different organismic demands and may prove helpful in monitoring responses to different exercise intensities, movement frequencies, and exercise durations. Additionally, this approach provides a more systemic view on cardiac autonomic regulation in the context of complex models of exercise physiology. In this context, non‐linear fluctuations of HRV may be seen as an outcome of the complex dynamic interplay of electro‐physiological, hemodynamic, and humoral variables, along with the effects of autonomic and central nervous system regulation. Thus, the measurement of non‐linear HRV during exercise and recovery might help gain further insights into the complex heart‐brain integration response as part of a general exercise related regulation capacity. The available data suggest that DFA‐alpha1 could be used as an adequate and easy‐to‐access load measure for exercise training. From the practical perspective of training and exercise science, it would be interesting to further investigate the influence of structured training, therapeutic interventions, and different performance levels on the described processes of cardiac autonomic regulation during exercise. These findings might lead to a broader understanding of the underlying mechanisms which, in turn, could open up possibilities of new strategies to evaluate and enhance the state of health, disease, or performance level.

## CONFLICT OF INTERESTS

The authors declare that the research was conducted in the absence of any commercial or financial relationships that could be construed as a potential conflict of interest.

## AUTHOR CONTRIBUTIONS

TG and OH conceived and designed the study details. Both authors performed the literature analysis and study selection. TG wrote the first draft of the manuscript. OH reviewed and edited the draft critically. Both authors read and accepted the final version of the manuscript.
